# Neonatal Enterovirus-Associated Myocarditis in Dizygotic Twins: Myocardial Longitudinal Strain Pattern Analysis

**DOI:** 10.3390/children11050506

**Published:** 2024-04-24

**Authors:** Stefania Giampetruzzi, Domenico Sirico, Nicoletta Mainini, Marta Meneghelli, Enrico Valerio, Sabrina Salvadori, Giovanni Di Salvo

**Affiliations:** 1 Neonatal Intensive Care Unit, Department for Women’s and Children Health, University of Padua, 35128 Padua, Italy; stefania.giampetruzzi@aopd.veneto.it (S.G.); nicoletta.mainini@aopd.veneto.it (N.M.); marta.meneghelli@aopd.veneto.it (M.M.); enrico.valerio@aopd.veneto.it (E.V.); sabrina.salvadori@aopd.veneto.it (S.S.); 2 Pediatric and Congenital Cardiology Unit, Department for Women’s and Children Health, University of Padua, 35128 Padua, Italy; giovanni.disalvo@unipd.it

**Keywords:** enterovirus, myocarditis, newborn, echocardiography

## Abstract

Enteroviruses (EVs) are the most common causes of viral myocarditis in neonates. Neonatal enterovirus myocarditis manifestations range from nonspecific febrile illness to congestive heart failure and cardiogenic shock with high risk of in-hospital mortality and long-term cardiac sequelae. Early recognition is essential to undertake appropriate therapy and predict outcomes. Echocardiography and echo-derived left ventricular strain measures seem promising for these purposes. We herein report two cases of neonatal enterovirus-associated myocarditis in dichorionic diamniotic twins, with different presentation, clinical course, and intensity of treatments.

## 1. Introduction

Enterovirus (EV) infections are common in children, and newborns are particularly at risk of severe infection [1,2]. An epidemiological survey of neonatal non-polio enterovirus infection in the Netherlands shows an incidence of EV infection in the neonatal period of 26 cases per 100,000 live births [2]. Neonates can acquire infection through vertical transmission before birth (transplacental), from exposure to maternal blood or secretions during delivery, or by horizontal transmission from family members or healthcare workers [3,4,5,6,7,8]. Different risk factors for severe disease in newborns are described: lack of neutralizing antibodies to the infecting serotype, maternal illness in the peripartum, prematurity, and onset of disease in the first week of life [5]. Enterovirus can be detected in serum, respiratory secretions, stool, and Cerebral Spinal Fluid (CSF), and RT-PCR (Reverse transcriptase–Polymerase Chain Reaction) is the most rapid, sensitive, and available molecular diagnostic method [5]. Clinical manifestations range from asymptomatic or nonspecific febrile illness to severe systemic involvement including myocarditis, meningoencephalitis, acute hepatitis, coagulopathy, and multiorgan failure [3,9,10,11,12,13,14]. When myocarditis occurs, it is often associated with disseminated infection and higher mortality [4,8,15,16,17]. Group B Coxsackieviruses are the most frequent causative agents of EV myocarditis [18,19]. In cases of myocardial involvement, the main treatment consists of symptomatic and supportive care [3,5,8]. In fact, the literature is still lacking regarding the use of intravenous immunoglobulin and experimental antiviral drugs (Pleconaril and Pocapavir) in neonates [14,20,21,22,23]. The prognosis is variable with a high mortality rate (30–50%) and significant risk of long-term cardiac injury. Nevertheless, about 50% of patients with enterovirus-induced myocarditis completely recover without residual injury [22,24,25,26]. Echocardiography is the primary diagnostic test that detects left ventricular systolic dysfunction and other abnormalities correlated with myocardial inflammation, but the utility of ultrasound indices in predicting outcomes in neonatal myocarditis, particularly myocardial strain imaging, has not been fully established yet [20]. Several studies demonstrated that 2D speckle-tracking-derived evaluation (2D STE) of myocardial strain may detect LV dysfunction prior to ejection fraction (EF) reduction [27,28,29,30]. As a consequence, performing STE in neonates infected with enterovirus without signs or symptoms of myocardial involvement could be of critical importance in the early detection of cardiac dysfunction and in the early initiation of anti-remodeling therapy.

In this study, we present the case of a pair of dizygotic twins infected by the same enterovirus serotype (Coxsackievirus B5) with myocardial involvement, yet with different clinical presentation and disease course severity.

## 2. Cases Presentation

Male and female dichorionic, diamniotic twins were born at 34 + 5 gestational weeks delivered by emergency caesarean section due to acute onset of maternal abdominal pain. The course of pregnancy until then was normal. Maternal serologies were protective. Group B Streptococcus (GBS) screening was not performed.

### 2.1. Case 1

**Figure 1:**
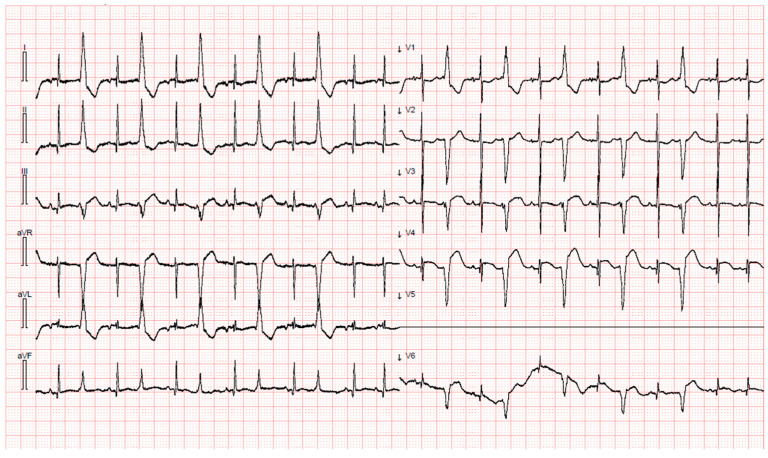
Case 1 EKG showing premature ventricular contractions in a pattern of ventricular bigeminy.

**Figure 2:**
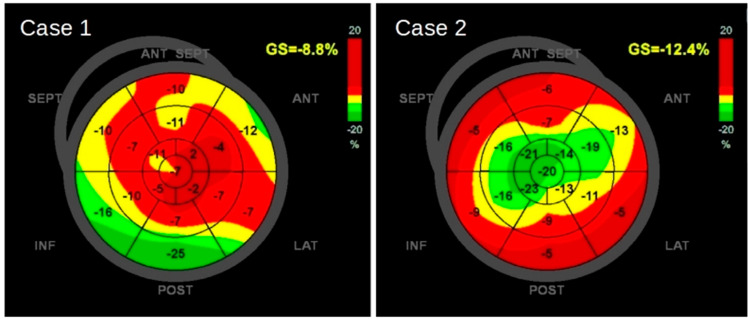
(**Right panel**) Twin 1 speckle-tracking evaluation (STE) at presentation showing reduced global left ventricular (LV) systolic function (EF 25%), with a severe reduction in global longitudinal strain (GLS −8.8%), and evidence of base-to-apex gradient. (**Left panel**) Twin 2 speckle-tracking evaluation (STE) at presentation showing mild LV systolic dysfunction (EF 45%) and reduced GLS (−12.2%) with basal segments more impaired compared to mid and apical ones.

Patient 1 was the male twin. At birth, his APGAR score was 6-7-8 at 1, 5, and 10 min, respectively. The birth weight was 2460 g. Continuous Positive Airway Pressure (CPAP) was started for respiratory distress. Because of increasing dependence on oxygen, a first dose of surfactant was administered through the INSURE method at 4 h of life. At 24 h of life, the infant presented a worsening of respiratory distress, and antibiotic therapy with ampicillin and netilmicin was started. A second dose of surfactant was administered followed by mechanical ventilation. After 48 h, the infant was extubated and non-invasive ventilation was embedded. On the seventh day of life (DOL), the persistence of respiratory distress and the occurrence of abdominal distension and diarrhea led to the suspicion of late-onset sepsis. Blood tests were performed and showed hemolysis without anemia, thrombocytopenia without significant bleeding, coagulopathy, and acute hepatitis (Table 1). CRP was 15 mg/L. Several viruses were PCR-tested in respiratory, blood, stool, and Central Nervous System (CNS) samples through the RT-PCR method. The results were all positive for enterovirus. Stool, Cerebral Spinal Fluid (CSF), and blood cultures were negative. The analysis of CSF was consistent with aseptic meningitis. The antibiotic therapy was initially shifted to oxacillin and ceftazidime and then discontinued on the basis of negative cultures. Platelet and plasma transfusion was performed, and the first dose (1 g/Kg) of intravenous immunoglobulin (IVIG) was administered based on the multisystemic presentation. Two days after the onset of infection (ninth DOL), the patient presented a decline in general conditions with pallor, hypotonia, hyporeactivity, and premature ventricular contractions in a pattern of ventricular bigeminy on EKG (electrocardiogram) monitor trace (Figure 1). Due to a suspicion of myocarditis, a heart ultrasound was performed and showed reduced global left ventricular (LV) systolic function (EF 25%), with a severe reduction in global longitudinal strain (GLS −8.8%) and evidence of a base-to-apex gradient, with the apical segments more affected compared to the mid and basal ones (Figure 2, Table 2). Blood tests showed significant elevation of hs-Troponin I (8741 ng/L) and NT-proBNP (31,350 ng/L). Inotropic support with milrinone and low-dose adrenaline and intravenous heparin prophylaxis were started. An additional dose (1 g/kg/d) of IVIG was administered. ECMO support was not needed. On the tenth DOL, electric seizures occurred and phenobarbital was introduced with the resolution of the amplified-EEG rhythmic pattern. Cerebral Magnetic Resonance Imaging showed multifocal white matter lesions of fronto-parietal regions. In the following days, the infant presented an improvement in general condition. He was gradually weaned from mechanical ventilation. The inotropic support was progressively discontinued and heart failure treatments with ACE inhibitors, diuretics, aldosterone receptor antagonists, and beta blockers were started. The last echocardiography before discharge showed improved cardiac function with an LV ejection fraction of 37%, moderate mitral valve regurgitation, and anterior-basal, inferior, and apical interventricular septal akinesis. Enteroviral serotyping and VP1 genotyping was performed and showed CVB5 infection.

### 2.2. Case 2

Patient 2 was the female twin. The infant was vigorous at birth with APGAR scores of 8-8-8 at 1, 5, and 10 min. The birth weight was 2325 g. She did not require intensive care and was discharged from the nursery on the fifth DOL. On the ninth DOL, the patient presented with an occurrence of diarrhea. Considering the clinical situation of her twin brother, she was admitted to our Neonatal Unit for further investigations. Echocardiography was performed and demonstrated mild LV systolic dysfunction (EF 45%) and severe GLS impairment (−12.2%) (Figure 2). However, conversely to the male twin, segmental strain analysis displayed major impairment of the basal segments of all left ventricle walls and interventricular septum, lateral, and posterior wall mid segments, while apical ones were relatively spared (Table 2). In the suspect of myocarditis, two doses of IVIG (2 g/Kg) were administered and anti-remodeling therapy with ACE inhibitors was started. She remained asymptomatic and did not require inotropic support. EKG was normal. Blood exams showed moderate elevation of hs-Troponin I (680 ng/L) and NT-proBNP (10,724 ng/L), as well as normal blood count, coagulation, and hepatic function. The maximum value of CRP was 10 mg/L (Table 1). RT-PCR confirmed EV in plasma, respiratory secretions, and stools. Blood and stool cultures were negative and antibiotic therapy was not started. Four days after her admission, the infant presented febrile illness associated with hyporeactivity. In the suspect of meningoencephalitis, a lumbar puncture was performed. The analysis of CSF was consistent with aseptic meningitis and EV was detected in the sample. The EEG and MRI were normal. Her general condition gradually improved and the last echocardiography before discharge (26th DOL) showed an LV ejection fraction of 54% and basal segment hypokinesia. Enteroviral serotyping and VP1 genotyping was performed and showed CVB5 infection.

## 3. Discussion

This study presents the case of a pair of newborn twins affected by EV infection, both developing myocarditis, although with different clinical courses of the disease. Case 1 and case 2 presented symptoms on the seventh and ninth DOL, respectively. Case 1 had multisystemic involvement (gastrointestinal symptoms, coagulopathy, hepatitis, myocarditis, and meningoencephalitis) and more severe disease compared to case 2. Interestingly, both cases developed myocarditis, yet with different clinical phenotypes and patterns of myocardial damage at echocardiographic examination.

Although EVs, especially Group B Coxsackieviruses 1–5, play a pivotal role in 25–35% of proven viral myocarditis, not all infants affected by EV infection develop myocarditis [18,19]. In fact, recent data suggest that viral myocarditis could be genetically predisposed [31,32], and genetic background might lead to worse outcomes (i.e., dilated cardiomyopathy or arrhythmogenic cardiomyopathy) [33]. Our case of siblings may support the hypothesis of a virus-triggered and genetically determined myocarditis, although they were dizygotic twins and did not share the same gene pool. Nonetheless, factors associated with more severe myocardial damage remain unknown.

The diagnosis of myocarditis in infants is challenging and relies on history, clinical findings, EKG, and elevation of cardiac biomarkers (i.e., troponin I and NT pro BNP). Non-invasive imaging tests are available (two-dimensional echocardiography and cardiac magnetic resonance, CMR) and contribute to the diagnosis; however, outcome prediction remains challenging [20,34,35]. CMR’s usefulness for the diagnostic work-up of pediatric patients with suspected myocarditis has been demonstrated [20,36,37]. Late gadolinium enhancement (LGE) typically involves the subepicardial and/or mid-wall layers of the myocardium [20,38,39]. Nevertheless, CMR should not be performed in a critically ill patient, requires sedation, and presents lower image quality in the neonatal age.

Conventional two-dimensional (2D) echocardiography is the first-line imaging technique under suspicion of acute myocarditis. However, it only assesses global and qualitative regional ventricular function and may not be sufficient for the diagnosis in the case of normal or pseudo-normal systolic function due to the compensation of hypokinetic segments by hyperkinetic ones [7]. For this reason, the echocardiographic assessment of myocardial injury should comprise the 2D speckle-tracking-derived evaluation (2D STE) of myocardial strain. STE is based on frame-to-frame tracking of “speckles” created by the interaction of ultrasonic waves and myocardium, in order to determine the measurement of LV deformation [40,41]. The systolic deformation of the left ventricle produces a shortening of the myocardial fibers and, consequently, a negative value of longitudinal strain. Several studies demonstrated that this imaging modality may detect subtle myocardial injury and sub-clinical LV dysfunction prior to ejection fraction (EF) reduction [27,28,29,30]. Moreover, myocardial strain changes in systolic or diastolic function may correlate with tissue pathology observed on biopsy or CMR. Interestingly, echo-derived left ventricular strain measures were shown to be abnormal and guided risk stratification in a population of 14 infants with enteroviral myocarditis and variable clinical phenotypes [27]. Nevertheless, the above-mentioned study was a retrospective analysis of GLS in a cohort of patients with clinically relevant enterovirus-related myocarditis, all previously diagnosed with systolic dysfunction on standard 2D echocardiography.

Accordingly, both our cases presented an impairment of LV global longitudinal strain values (normal value below −20%), although this was worse in case 1 compared to case 2 (GLS −8.8% vs. −12.2%). It is interesting to note that case 2 was clinically asymptomatic at the onset of infection but showed a reduction in both GLS and LVEF, albeit milder than case 1. Furthermore, strain analysis showed opposite sites of cardiac damage between the two cases, with the apical segments more affected in case 1 versus the basal ones in case 2. This finding may suggest the absence of a virus preferential pattern of myocardial segment involvement. Finally, the evidence of severe GLS impairment in case 2 guided the prompt initiation of IVIG and anti-remodeling therapy.

Considering these factors, performing standard 2D and speckle-tracking echocardiography with left ventricle longitudinal strain analysis at the onset of EV infection, even in infants without signs or symptoms of cardiac involvement, might guide clinicians toward the early detection of mild or initial forms of myocarditis, and to start supportive and anti-remodeling therapy as soon as possible. Furthermore, speckle-tracking echocardiography would be helpful during the follow-up of these patients in order to monitor myocardial function and evaluate potential long-term myocardial injury recovery.

## 4. Conclusions

Enterovirus infection-related myocarditis shows a genetic predisposition. However, factors associated with worse heart involvement remain unknown. Echo-derived left ventricle strain analysis may help in identifying subjects with subclinical myocardial injury. Early recognition of myocardial damage might suggest the initiation of anti-remodeling therapy and guide cardiologic follow-up.

## Figures and Tables

**Table 1:** Laboratory tests at disease onset.

	Case 1	Case 2
Leucocytes (10⁹/L)	23,640	10,770
Neutrophils (10⁹/L)	10,710	5050
Hemoglobin (g/dL)	16.1	13.6
Platelets (10⁹/L)	8000	221,000
CRP (mg/L)	14.18	0.62
INR ratio	1.18	1.02
aPTT ratio	1.60	1.14
D-dimer (µg/L)	25,839	6422
FDPs (g/L)	0.72	1.95
AST (U/L)	307	35
ALT (U/L)	126	22
GGT (U/L)	113	37
Total bilirubin (µmol/L)	62.6	39.9
Conjugated bilirubin (µmol/L)	11.4	7.8
hs-Troponin I (ng/L)	7543	680
NT-proBNP (ng/L)	31,353	10,724

Legend: CRP, C-Reactive Protein; INR, International Normalized Ratio; aPTT activated Partial Thromboplastin Time; FDPs: Fibrinogen Degradation Products; ALT, Alanine Aminotransferase; AST, Aspartate Aminotransferase; GGT: Gamma-Glutamyl Transferase; NT pro BNP: N-terminal pro-brain natriuretic peptide.

**Table 2:** LV wall segment quantitative longitudinal strain analysis.

LV Segmental LS	Case 1	Case 2
LV A4C LS (%)		
LV basal posterior septum	−10	−5
LV mid posterior septum	−11	−16
LV apical posterior septum	−5	−21
LV apical lateral	−3	−13
LV mid lateral	−7	−11
LV basal lateral	−7	−5
LV A2C LS (%)		
LV basal inferior	−16	−9
LV mid inferior	−10	−16
LV apical inferior	−6	−22
LV apical anterior	−4	−14
LV mid anterior	−4	−19
LV basal anterior	−12	−13
LV A3C LS (%)		
LV basal posterior	−25	−5
LV mid posterior	−7	−9
LV apical posterior	−4	−14
LV apical antero-septal	−7	−18
LV mid antero-septal	−11	−7
LV basal antero-septal	−10	−6
LV average GLS (%)	−8.8	−12.4

Legend: A4C, apical four-chamber view; A2C, apical two-chamber view; A3C, apical three-chamber view; GLS, global longitudinal strain; LS, longitudinal strain; LV, left ventricle.

## Data Availability

The data presented in this study are available on request from the corresponding author. The data are not publicly available due to privacy restrictions.
